# Oscillating Magnet Array−Based Nanomagnetic Gene Transfection: A Valuable Tool for Molecular Neurobiology Studies

**DOI:** 10.3390/nano7020028

**Published:** 2017-01-29

**Authors:** Mahendran Subramanian, Aimee-Jayne Tyler, Eva Maria Luther, Elena Di Daniel, Jenson Lim, Jon Dobson

**Affiliations:** 1 nanoTherics Limited, Keele University Science Park, Keele ST5 5NL, UK; aimeejayne@nanotherics.com (A.-J.T.); eva@nanotherics.com (E.M.L.); 2 Takeda Cambridge, 418 Cambridge Science Park Milton Rd, Milton, Cambridge CB4 0PZ, UK; elena.didaniel@ndm.ox.ac.uk; 3 Biological and Environmental Sciences, School of Natural Sciences, University of Stirling, Stirling FK9 4LA, UK; jenson.lim@stir.ac.uk; 4 J. Crayton Pruitt Family Department of Biomedical Engineering, Department of Material Science and Engineering, and Institute for Cell Engineering and Regenerative Medicine—ICERM, University of Florida, P.O. Box 116131, Gainesville, FL 32611, USA; jdobson@bme.ufl.edu

**Keywords:** magnetic nanoparticles, transfection, SH-SY5Y, hippocampal neurons, cortical neurons, non-viral gene delivery, nanomagnetic gene transfection, biomagnetics, gradient magnetic field, permanent magnets

## Abstract

To develop treatments for neurodegenerative disorders, it is critical to understand the biology and function of neurons in both normal and diseased states. Molecular studies of neurons involve the delivery of small biomolecules into cultured neurons via transfection to study genetic variants. However, as cultured primary neurons are sensitive to temperature change, stress, and shifts in pH, these factors make biomolecule delivery difficult, particularly non-viral delivery. Herein we used oscillating nanomagnetic gene transfection to successfully transfect SH-SY5Y cells as well as primary hippocampal and cortical neurons on different days in vitro. This novel technique has been used to effectively deliver genetic material into various cell types, resulting in high transfection efficiency and viability. From these observations and other related studies, we suggest that oscillating nanomagnetic gene transfection is an effective method for gene delivery into hard-to-transfect neuronal cell types.

## 1. Introduction

Neurons are key units of the central nervous system (CNS) which process and transmit information through electrical and chemical signalling [1,2]. Dysfunction of these critical physiological processes can lead to a host of neurological diseases such as Alzheimer’s disease (AD) [3], Parkinson’s disease (PD) [4], epilepsy [5], and multiple sclerosis [6]. To better understand the biology and function of neurons in the brain, current research involves the delivery of small biomolecules such as DNA, RNA or peptides into cultured neurons by means of transfection to enhance or silence the expression of genes or proteins [7,8,9,10,11]. However, primary neurons in culture are very sensitive to temperature changes, stress, and pH changes [12,13,14], which creates problems for the delivery of small biomolecules [15]. 

Hence, neuronal cell lines are used, for example SH-SY5Y, which is a cell line derived from human neuroblastoma, which can be differentiated into neuronal-like cells with properties of adult neurons [16,17]. SH-SY5Y has served as a model for studying neuron biology, especially in neurodegenerative diseases such as AD and PD [18,19,20], and transfection experiments have been conducted on SH-SY5Y to understand neuron damage and degeneration [21], growth and regeneration [22], and the role of nuclear location sequences in non-viral transfection [23]. 

Herein, we demonstrate the successful transfection of the cell line SH-SY5Y, along with primary rat hippocampal and cortical neurons, using magnetic nanoparticles (MNPs) and an oscillating magnet array with no impact on cell viability. 

## 2. Results

### 2.1. Magnetostatic Calculation

**Figure 1:**
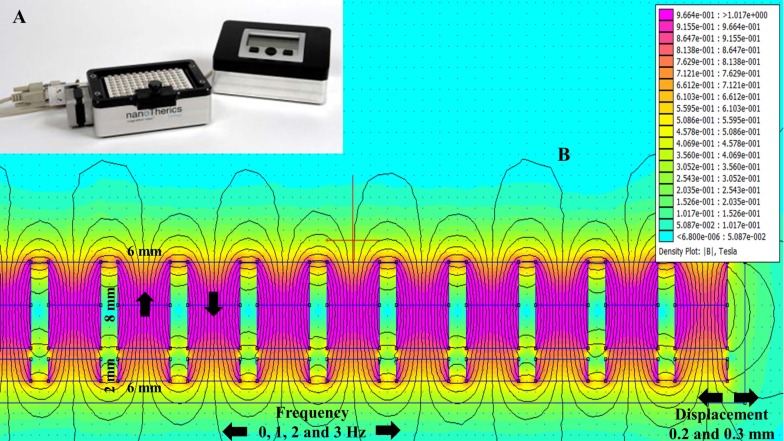
Oscillating magnet array−based nanomagnetic gene transfection experimental setup. (**A**) Representation of a 96-well oscillating magnet array–based nanomagnetic transfection setup using NdFeB magnetic array; (**B**) Dimensions of the permanent magnets and magnetostatic (vector-potential) algorithm based magnetic field density |B| distribution (T) contour plot for the NdFeB magnetic array.

The oscillating magnet array, shown in Figure 1A, is made up of a 96-well alternate pole magnet array that oscillates in a horizontal/lateral motion at a pre-determined displacement and frequency. While the use of an oscillating magnet array has been shown to successfully transfect a range of cell types with little detrimental effect on cell viability [24,25,26,27,28,29,30], this study determined whether this method can be used to successfully transfect sensitive neuronal cell lines and primary neurons. 

**Figure 2:**
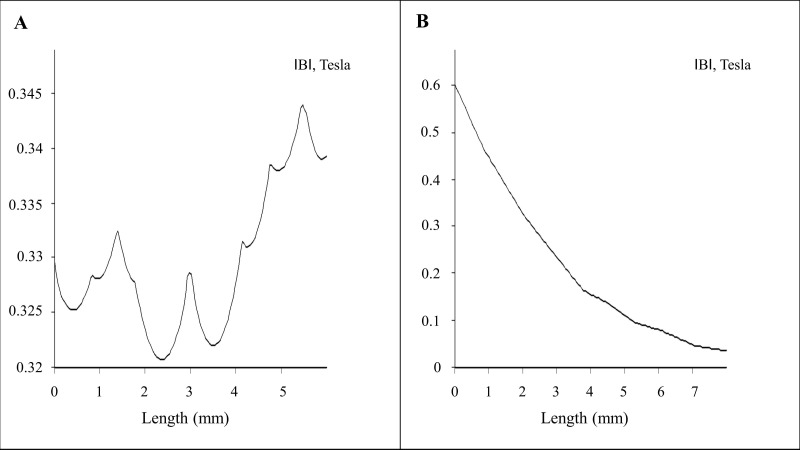
Magneto-static (vector potential) algorithm–based numerical calculations: (**A**) 2D plot of the magnetic field for *x* axis (marked in red line horizontal above the magnet array in Figure 1B); (**B**) 2D plot of the magnetic field for *y* axis (marked in red line vertical above the magnet array in Figure 1B).

Finite element method magnetics (FEMM)-based magneto static calculation data along with a magnetic field density |B| (Tesla) distribution contour plot are provided in Figure 1B. The magnetic field density |B| on the *x* axis and *y* axis (marked with the red line above the magnet in Figure 1B) over a distance is provided in Figure 2A,B, respectively. The magnetic field amplitude for the magnet array is 320 ± 25 mT and the field gradient ranges between 100–200 T/m (center to edge of the well) at the cell surface.

### 2.2. Transfection of Undifferentiated SH-SY5Y Cells

**Figure 3:**
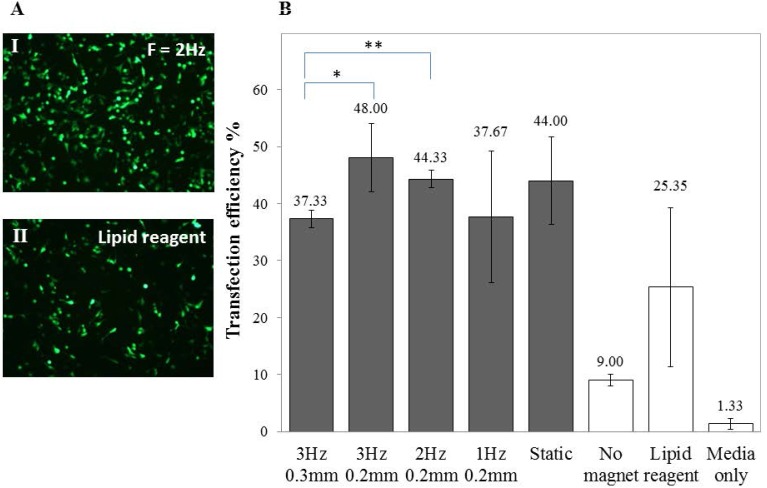
Higher gene expression in undifferentiated SH-SY5Y cells. (**A**) Fluorescent images of GFP-expressing SH-SY5Y cells transfected using (I) an oscillating magnet array (Frequency = 2 Hz; Displacement = 0.2 mm) or (II) a cationic lipid–based reagent; (**B**) Bar chart showing the percentage of GFP-expressing cells 48 h after transfection with different oscillating magnet array settings. FACS data shown are the mean ± SD of (*n* = 3), respectively.

To optimise the transfection of SH-SY5Y cells, various Green Fluorescent Protein (GFP) to Magnetic Nanoparticles (MNP) ratios (GFP: MNP), frequencies and displacements of the magnet array were investigated. A ratio of 1:1 of GFP to PolyMag was found to give the maximum transfection results (data not shown) and the transfection efficiency varied when subjected to an oscillating magnet of varying frequency, as demonstrated in Figure 3. Viability for SH-SY5Y cells (3 Hz, 0.2 mm; 0.2 µL PolyMag: 0.2 µg DNA) was 82.33% ± 3.88%.

### 2.3. Gene Delivery and Prolonged Expression in Primary Hippocampal Neurons on Different Days In Vitro

**Figure 4:**
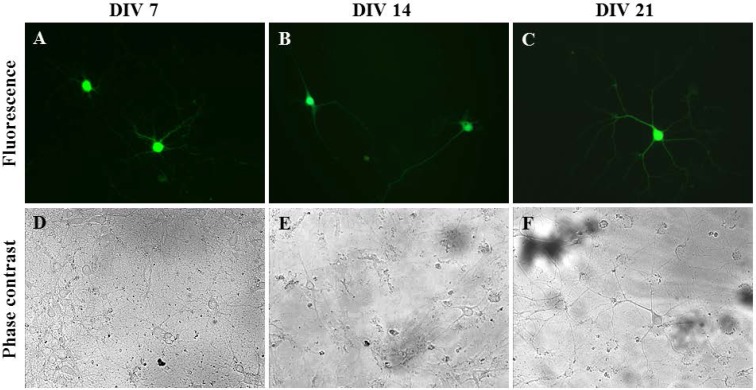
Neuron-specific gene delivery methods in primary hippocampal cells. Images of Synap 1 plasmid expressing primary neurons (2 × 10^4^ cells/well). Fluorescent and phase contrast images of transfected DIV 7 (**A**,**D**), DIV 14 (**B**,**E**) and DIV 21 (**C**,**F**) mature neurons using an oscillating magnet array (Frequency = 2 Hz; Displacement = 0.2 mm), imaged at 96 h (**A**,**D**) or 48 h (**B**,**C**,**E**,**F**) post transfection.

To ensure that only primary neurons were transfected within the disassociated hippocampal tissue, Synap 1, a plasmid with a GFP cassette that is driven by the neuron-specific Synapsin I (SYNI) promoter, was used [31]. Rat hippocampal neurons isolated on different days in vitro, i.e., Days In Vitro (DIV) 7, DIV 14 and DIV 21, were successfully transfected by oscillating nanomagnetic gene transfection without damaging the neurite growth, as seen in Figure 4. The high level of GFP expression persisted up to 48 h (Figure 4B,C,E,F) or 96 h (Figure 4A,D) in primary hippocampal neurons. The transfection efficiency and viability for primary hippocampal neurons were 15% ± 5.00% and 75.00% ± 5.00% (*n* = 3), respectively.

### 2.4. Gene Delivery by Oscillating Nanomagnetic Gene Transfection in Primary Cortical Neurons

**Figure 5:**
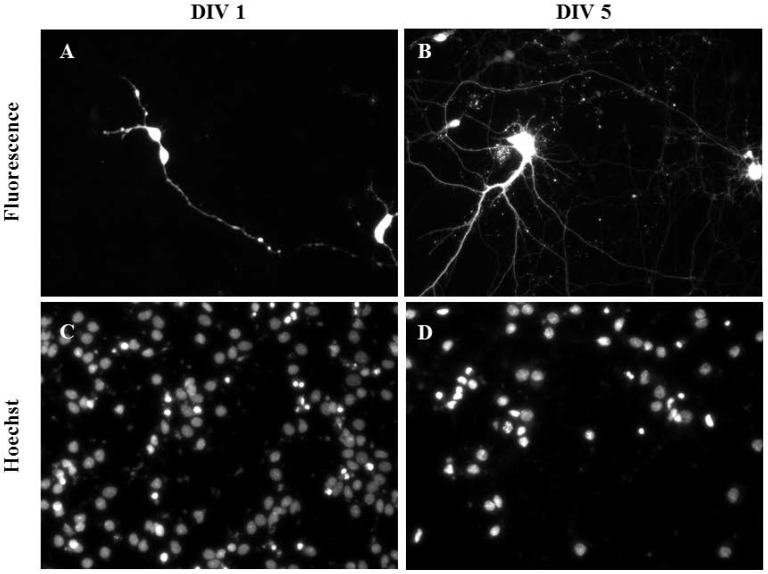
Gene delivery by oscillating nanomagnetic gene transfection in primary cortical neurons. Images of pmaxGFP plasmid expressed in primary neurons using fluorescence microscopy and its corresponding Hoechst 33,342 stained counterpart of transfected DIV 1 (**A**,**C**) and DIV 5 (**B**,**D**) mature neurons were taken 48 h post transfection.

Primary cortical neurons were also successfully transfected as demonstrated using mature cortical neurons transfected at DIV 1 (Figure 5A,C) and DIV 5 (Figure 5B,D). The transfection efficiency and viability (2 Hz, 0.2 mm; 0.1 µL NeuroMag, 0.05 µg DNA) of primary cortical neurons were 10.00% ± 5.00% and 75.00% ± 5.00% (*n* = 3), respectively. Controls containing DNA only and NeuroMag only showed no transfection and their viability was comparable to the control containing media only (data not shown).

## 3. Discussion

Previously, the combination of nanomagnetic and lipid reagent–based gene transfection demonstrated advantages such as cost efficiency, minimised complexity (unlike viral constructs), long-term gene expression and the ability to transfect various plasmid constructs in both young and mature neurons (from DIV 5 to DIV 21) [7]. Recently, motor neurons [8], cortical neurons [32] and cerebellar granule neurons [33,34] were transfected using NeuroMag, though most studies focused on individual cells. An alternative and well-characterised method is the use of the neuronal cell line SH-SY5Y, which is able to undergo differentiation from precursor cells to neuroblastoma cells [22,35,36,37].

In this study we investigated the impact of an oscillating magnetic field on magnetic nanoparticles coupled to plasmid DNA during the transfection of both the neuron-like cell line SH-SY5Y and primary neurons. Oscillating nanomagnetic transfection improved the transfection efficiency and expression of the reporter protein with low cytotoxicity and non-interference with the cell physiology, such as differentiation and neurite growth, in both cell lines and primary neurons. These results are similar to studies on other cell types using the same technology [23,24,25,26,27,28,30,38,39,40]. It is also interesting to note that this oscillating nanomagnetic gene transfection can be used for transfecting rat hippocampal neurons with a large plasmid, leading to a prolonged and high gene expression [41]. 

While the increase in transfection efficiency using the oscillating magnet array at different frequencies was modest (compared to a static magnet array), this was unsurprising as we have observed it in astrocytes [27] and adult cardiomyocytes [24]. However, we and others have shown that this is highly dependent on the cell type. Neural stem cells [40], oligodendrocyte precursor cells [30], cardiac progenitor cells [24], human umbilical vein endothelial cells (HUVEC) [26], mouse embryonic fibroblasts (MEF) [26], MG-63 osteoblasts [38], mesenchymal stem cells (MSC) [39] and astrocytes [27] all showed a frequency-dependent increase in transfection efficiency.

Furthermore, we do not yet have an explanation for the underlying biophysical interaction between the surface-functionalised magnetic nanoparticles and the oscillating magnet field due to particle agglomeration, the magnetic susceptibility of surface-functionalised nanoparticles and the viscous damping phenomenon (data not shown). However, magnetic nanoparticles will experience torque in a homogenous magnetic field, but will undergo translational movement when exposed to a field gradient. In addition, the field gradient is influenced by the magnet array displacement [42]. We propose two possibilities to explain the high gene expression. There is either a high level of plasmid DNA being introduced per cell, leading to a higher level of protein expression, or the DNA-magnetic nanoparticle complexes have redirected the cell machinery towards higher protein production. 

Oscillating nanomagnetic gene transfection has clinical potential, as shown in the normal expression of stem cell markers and axon/neurite growth in transfected and differentiated neural stem cells and dorsal root ganglion neurons, respectively [40,41]. Furthermore, transfected oligodendrocyte stem cells are not affected in migration, division and integration after transplantation into a 3 dimensional tissue culture model [30]. 

## 4. Materials and Methods 

### 4.1. Materials

Hoechst 33342, antibiotics or other media supplements were purchased from Sigma (Dorset, UK). Lipofectamine 2000 from Invitrogen (Paisley, UK) and all cell culture plastics were from Costar (Appleton Woods (Birmingham, UK). Hams F12, MEM, neuro basal medium, foetal bovine serum (FBS), other kits and all other chemicals of the highest purity available were from either Neuromics (Edina, MN, USA) or Biosera (East Sussex, UK). PolyMag and NeuroMag are magnetic nanoparticles surface functionalised with transfection-enhancing materials and contains a magnetite core. Both were from Ozbioscience (Marseilles, France). PolyMag is 100–250 nm in hydrodynamic diameter and has been successful in transfecting SH-SY5Y cells [23]. PolyMag is surface functionalised with a proprietary polyethylenimine derivative with positive zeta potential [39,43,44,45]. 

NeuroMag is 140–200 nm hydrodynamic diameter in size [27,46] and was chosen for transfecting primary hippocampal and cortical neurons as it has been used successfully in transfecting astrocytes [27], cerebellar granule cells [33], dorsal root ganglia [47], and motor neurons [8]. Like PolyMag, it has a proprietary coating that is positively charged [27]. The rationale for using both particles is that the study was aimed at evaluation of magnet array technology. Our goal was to demonstrate that the technology works efficiently with different commercially available magnetic nanoparticles that have been widely used to transfect neurons, rather than using NeuroMag alone.

### 4.2. Cell Culture 

Human neuroblastoma cells (SH-SY5Y) (No. CRL-2266; American Type Culture Collection, Manassas, VA, USA) were maintained as previously described [48]. SH-SY5Y were seeded at a density of 1 or 2 × 10^4^ cells in 100 µL medium onto 96-well plates in Ham’s F12:MEM (1:1) supplemented with 10% FBS, 2 mM l-glutamine, 100 U penicillin and 0.1 mg/mL streptomycin. Both cell lines were incubated at 37 °C, 5% CO_2_, for 24 h before performing transfection experiments. Primary rat hippocampal and cortical neurons were obtained from Neuromics (Edina, MN, USA) and disassociated using papain disassociation kit (Worthington, NJ, USA) according to the manufacturer’s instructions. Isolated neurons were maintained using neurobasal medium supplemented with 5% FBS, 0.5 mM Glutamax, 2% B27 supplement, 25 µM l-glutamine and seeded onto poly-d-lysine–coated cells culture plates. 

### 4.3. DNA Constructs

Eukaryotic expression vectors pEGFP-N1 (4.7 kb) and pmaxGFP (3.5 kb) contain cytomegalovirus (CMV) promoters which drive overexpression for the gene encoding green fluorescence protein, and are purchased from Clontech (Mountain View, CA, USA) and Lonza (Slough, UK), respectively. Synap 1 (11 kb) and Neuron specific SYN1 promoter driven gene encoding green fluorescent protein were donated by NIH, USA. Plasmid DNA was prepared using the Qiagen Endo Free Plasmid Purification kit (Qiagen, Crawley, UK), and maintained in endonuclease-free water (Sigma, Dorset, UK) at −80 °C.

### 4.4. Transfection of Neurons (CN) 

#### 4.4.1. Nanomagnetic Gene Transfection 

To prepare transfection complexes, 0.3 or 0.05 μg pEGFP-N1, respectively, were diluted in 20 µL serum-free media, added to 0.3 µL PolyMag or 0.15 µL NeuroMag and mixed. After 20 min, complexes were added drop-wise to cells; the ratio of DNA: MNP in these experiments were 1:1 for SH-SY5Y. The final concentration of NeuroMag for neuron experiments was 0.1 µL per well (0.7 µL/mL medium); this concentration found to have no effect on cell viability [27]. Control wells comprised of un-transfected cells. An alternate pole magnet array for 96-well tissue culture plate was used for both static and oscillating transfection. For oscillating transfections, the magnet array was controlled using the Magnefect-nano II system (nanoTherics, Stoke-on-Trent, UK) as shown in Figure 1A. The frequency of oscillation was between 1–3 Hz with displacement between 0.2–0.3 mm. Plates were incubated in the presence of magnetic field conditions for 30 min. After transfection, cells were incubated for 48 h at 37 °C, 5% CO_2_ before analysis. 

#### 4.4.2. Lipid-Based Transfection 

Lipid-based transfections were performed in serum-free medium using 0.1 µg of pEGFP-N1 and 0.3 µg of Lipofectamine 2000 per well following the manufacturer’s recommended protocol. Mock-transfection or serum free medium were used as controls. After transfection cell culture plates were transferred back to the incubator for 48 h before analysis.

### 4.5. Flow Cytometry

Transfected cells were washed in 0.5% bovine serum albumin and phosphate-buffered saline (PBS) and analysed for the relative fluorescence of gated cells, using a FACSort analyser (Becton, Dickinson and Company, Oxford, UK). Transfection efficiency was determined by the percentage of gated cells in the FL1 channel over the total number of gated cells. 

### 4.6. Fluorescent Microscopy 

GFP expression in cells was analysed by microscopy using a fluorescent microscope (IX71-Olympus, Essex, UK). A minimum of three images were captured at fixed exposure settings, and an average percentage of the proportion of GFP-expressing cells related to the total number of cells was determined (transfection efficiency) using Image J (NIH, Bethesda, MD, USA).

### 4.7. Cell Viability Assay

CytoTox-ONETM homogenous membrane integrity assay (Promega, Southampton, UK) provided the measure of released lactate dehydrogenase (LDH) through damaged membrane of dead cells and the assay was performed according to manufacturer’s instruction. Luminescence was recorded using a plate reader (BioTek, Bedfordshire, UK). Viability data is expressed as a percentage using the following formula: Percentage viability = 100 − (100 × (Experimental − Background Luminescence)/ (Maximum LDH release − Background Luminescence)).

### 4.8. Statistics

If not stated otherwise, the data are presented as means ± SD of values from at least three experiments. Analysis of the significance of the differences between groups of data was performed by one-way ANOVA followed by Bonferroni post hoc test (multiple comparison test), *p* > 0.05 was considered as not significant.

### 4.9. Numerical Model and Magnetic Field Calculation 

The magnetic field |B| numerical calculations were performed using Finite Element Method Magnetics (FEMM 4.32, Waltham, MA, USA). In order to model magnetic field density |B| distribution from the permanent magnets, i.e., alternate pole magnet array arranged in a 96-well tissue culture plate format. We have applied the magnetostatic solution solver algorithm via the Finite Element Method model with Asymptotic Boundary Condition (ABC) conditions. In our case the material was Neodymium-Iron-Boron (NdFeB) with an energy product of 42 megaGauss Oersteds (MGOe). Dimensions of the permanent magnets can be seen in Figure 2B. 

## 5. Conclusions 

We were able to successfully transfect neuronal cells using this novel technique with little/no detrimental effect on the viability. This is key to effective in vitro and in vivo gene delivery in a physiological and clinical setting and further multidisciplinary research is required to make substantial progress in this area.
